# Zinc Is Probably Not Accumulated as Zinc Sulfide, and Is Co-Released with Glutathione to the Culture Medium, in the Marine Alga *Ulva compressa*

**DOI:** 10.3390/ijms27198827

**Published:** 2026-10-02

**Authors:** Matias Morales, Stephanie Romero, Patricia Méndez, Rodrigo Segura, Alberto González, Alejandra Moenne

**Affiliations:** 1 Laboratory of Marine Biotechnology, Faculty of Chemistry and Biology, University of Santiago of Chile, Alameda 3363, Santiago 917022, Chile; 2 Laboratory of Inorganic Chemistry, Faculty of Chemistry and Biology, University of Santiago of Chile, Alameda 3363, Santiago 917022, Chile

**Keywords:** zinc accumulation, sulfur donor, glutathione, phytochelatins, metallothioneins, *Ulva compressa*

## Abstract

The mechanisms involved in zinc (Zn) accumulation in response to a sulfur donor (NaHS) and a sulfur acceptor (hypotaurine) were analyzed in the marine alga *Ulva compressa*. The alga was cultivated with 10 μM of Zn, with 10 μM of Zn and increasing concentrations of NaHS for 0 to 7 days, and with 10 μM of Zn and 500 μM of hypotaurine for 5 days. The levels of glutathione (GSH) and phytochelatins (PCs) were also analyzed as well as the levels of transcripts encoding metallothioneins (MTs). Results showed that the level of intracellular Zn mainly decreased in response to 50, 100 or 200 μM of NaHS, compared with the control treated with 10 μM of Zn, and decreased in response to hypotaurine. The level of GSH, PCs (PC2, PC3 and PC4) as well as the level of transcripts encoding MTs did not parallel the level of intracellular Zn, in response to Zn alone or Zn with a sulfur donor. Zn and GSH were released to the culture medium; their release did not increase in response to a sulfur donor, and PCs were not detected in the culture medium. Thus, zinc is probably not accumulated as Zn sulfide (ZnS) and it is co-released to the culture medium with GSH in the marine alga *U. compressa*.

## 1. Introduction

Plants are sessile organisms that are exposed to environmental stresses that can be classified as abiotic and biotic stresses. Plants require nutrients to obtain C, N, S and P and oligo-elements such as iron (Fe), zinc (Zn) and copper (Cu) in trace amounts because these elements are required for the function of proteins and enzymes [1]. Zn is an essential heavy metal that is required for the function of several proteins and enzymes such as zinc finger-containing transcription factors, DNA and RNA polymerases, Cu/Zn superoxide dismutase, cytochrome c oxidase, carbonic anhydrase, alcohol dehydrogenase, aldehyde dehydrogenase, glutamate dehydrogenase and carboxypeptidases, among others [1]. In eukaryotes more than 1000 proteins, which represent around 9% of total proteins, require Zn as a cofactor [2]. Zn is also essential for tryptophan synthesis that is essential to the production of the phytohormone auxin indole 3-acetic acid (IAA), and for chlorophyll synthesis [2]. Interestingly, ZnO nanoparticles protect the plasma membrane and organellar membranes from heat by activating the antioxidant system [3]. In addition, Zn deficiency can inhibit growth and protein synthesis and induce leaf chlorosis [2]. It is important to mention that Zn excess is toxic to plants, inducing oxidative stress that leads to the destruction of plasma and organellar membranes, finally triggering plant death [4].

Regarding Zn absorption in plants, Zn is mostly found in roots, but small amounts are detected in stems and shoots [2]. Most plants contain 30 μg to 100 μg of Zn g^−1^ of dry tissue (DT) but there are zinc hyperaccumulator plants like Brassicaceae that can display 10,000 μg of Zn g^−1^ of DT without any sign of toxicity. In general, above 300 μg of Zn g^−1^ of DT is considered detrimental to terrestrial plants [5]. Plants acquire Zn from the soil through the roots and most agricultural soils contain 30 to 100 μg of Zn g^−1^ of soil [6]. The translocation of Zn from roots to shoots occurs in the xylem, and this process requires active transporters such as ATP-dependent HMA, ZIP, IRT, NRAMP and YSL transporters for entry into cells, diffusion facilitators (CDF) family, and/or HMA family transporters for entry into and accumulate in the vacuole [2].

In plant cells, Zn can bind to glutathione (GSH), a tripeptide constituted by the amino acids glutamate, cysteine and glycine, that is synthesized by the enzymes γ-glutamyl-cysteine synthase (GCS) and glutathione synthase (GS), both of which require ATP [7]. Zn can also bind to phytochelatins (PCs) that are formed by condensed units of GSH and synthesized by the enzyme phytochelatin synthase (PCS). Plants and algae mainly synthesize PC2, PC3 and PC4 [8,9]. Zn can also bind to metallothioneins (MTs) that are small proteins (around 10 kDa) that are rich in cysteines (around 30% of total amino acids), and GSH, PCs and MTs bind Zn through the sulfhydryl groups of cysteines [10]. Regarding GSH and Zn, it has been shown that *Brassica napus* cultivated under hydroponic conditions containing normal Zn concentrations and treated with GSH on leaves showed an increased Zn loading into the xylem and a higher Zn concentration in the shoots, but it did not increase the accumulation of iron or manganese in the shoots, indicating a strong connection between Zn and GSH [10]. In addition, transgenic *Arabidopsis thaliana* plants overexpressing the enzymes GCS or GS showed an increased zinc loading into the xylem and the accumulation of Zn in the shoots, under a normal zinc supply and in response to GSH applied to the leaves [11]. PCs are also essential for Zn accumulation because *Arabidopsis* plants mutated in PCS1 did not tolerate normal or excessive amounts of zinc in the soil, showing an inhibition of growth and chlorosis [12].

Regarding metallothioneins and Zn accumulation, it has been shown that excess of Zn increased the expression of MT1a in rice; the overexpression of MT1a alleviated inhibition of growth and chlorosis induced by Zn excess leading to an increased tolerance to drought [13]. In rice, MT2b and MT2c were increased, and single and double mutants of these MTs showed a decreased level of Zn in the panicle and decreased grain yield; both MTs showed Zn-binding abilities when expressed in yeast [14]. In addition, H_2_S, a sulfur donor, increased tolerance to Zn excess in the Cd/Zn hyperaccumulator *Solanum nigrum* by reducing Zn uptake in the roots, by increasing the expression of zinc uptake transporters ZRT, IRT and NRAMP, and by increasing the expression of antioxidant enzymes superoxide dismutase (SOD), catalase (CAT) and ascorbate peroxidase (AP) and, in particular, the expression and amount of MT1a [15].

The marine alga *Ulva compressa* (Chlorophyta) is a cosmopolitan green macroalga that accumulates copper (Cu), cadmium (Cd) and Zn [16,17]. It was initially hypothesized that Cu, Cd and Zn were accumulated through binding to GSH, PCs and/or MTs, as it has been observed in plants [14,15,16]. However, using Transmission Electron Microscopy (TEM) coupled with Energy-Dispersive X-Ray Spectrometry (EDXS), it was demonstrated that Cu accumulates as electron-dense nanoparticles that contained Cu and sulfur (S) but not nitrogen (N) in the chloroplast [18]. In addition, a sulfur donor (NaHS) increases the accumulation of intracellular Cu, and Cu accumulates as electron-dense nanoparticles of CuS in the cytoplasm and in the chloroplast. These electron-dense nanoparticles contained Cu and S, but not N, and in higher amounts compared with the alga treated without the sulfur donor [18]. Furthermore, the alga treated with Cu released the metal to the culture medium with GSH, and not with PCs [15]. Thus, Cu is accumulated as CuS, and Cu ions are released to the culture medium with GSH in *U. compressa*.

On the other hand, the alga treated with cadmium (Cd) showed electron-dense nanoparticles that contained cadmium and O, but not S or N, suggesting that Cd is accumulated as CdO and/or bound to fatty acids that are stained by uranyl and osmium [19]. The alga treated with Cd and the sulfur donor NaHS showed an increased accumulation of cadmium and electron-dense nanoparticles containing Cd and O, but also S and N. Thus, the alga treated with a sulfur donor contained CdO, CdS, and probably Cd bound to GSH and/or PCs. In addition, the alga treated with Cd released the metal to the culture medium with GSH and PCs, and the sulfur donor increased the release of Cd, GSH and PCs to the culture medium [19]. Thus, Cd is accumulated as CdO and/or bound to fatty acids and is also extruded to the culture medium with GSH and PCs in *U. compressa*. Therefore, the mechanisms of Cu and Cd accumulation *U. compressa* are different; in both cases, their accumulation is increased by a sulfur donor, and these metals are released with GSH to the culture medium, and in the case of Cd also with PCs [19]. It is important to highlight that the release of Cd, GSH and PCs to the culture medium is increased by the sulfur donor, and that this process has not been analyzed in the case of Cu.

In this work, we analyzed the mechanism involved in Zn accumulation in *U. compressa*. To this end, we analyzed the intracellular level of Zn in the alga treated with 10 μM of Zn for 0 to 7 d, with 10 μM of Zn and increasing concentrations of a sulfur donor (NaHS) for 0 to 7 d, and with 10 μM of Zn and a sulfur acceptor (hypotaurine) for 5 d. Furthermore, we determined the levels of GSH and PCs and the expression of MTs to analyze if their levels correlate with the level of intracellular Zn or levels of GSH and PCs, or with the expression of MTs. Finally, we analyzed whether Zn is extruded to the culture medium with GSH and/or PCs, and how this process responds to a sulfur donor.

## 2. Results

### 2.1. Intracellular Accumulation of Zn in Response to a Sulfur Donor or a Sulfur Acceptor

**Figure 1:**
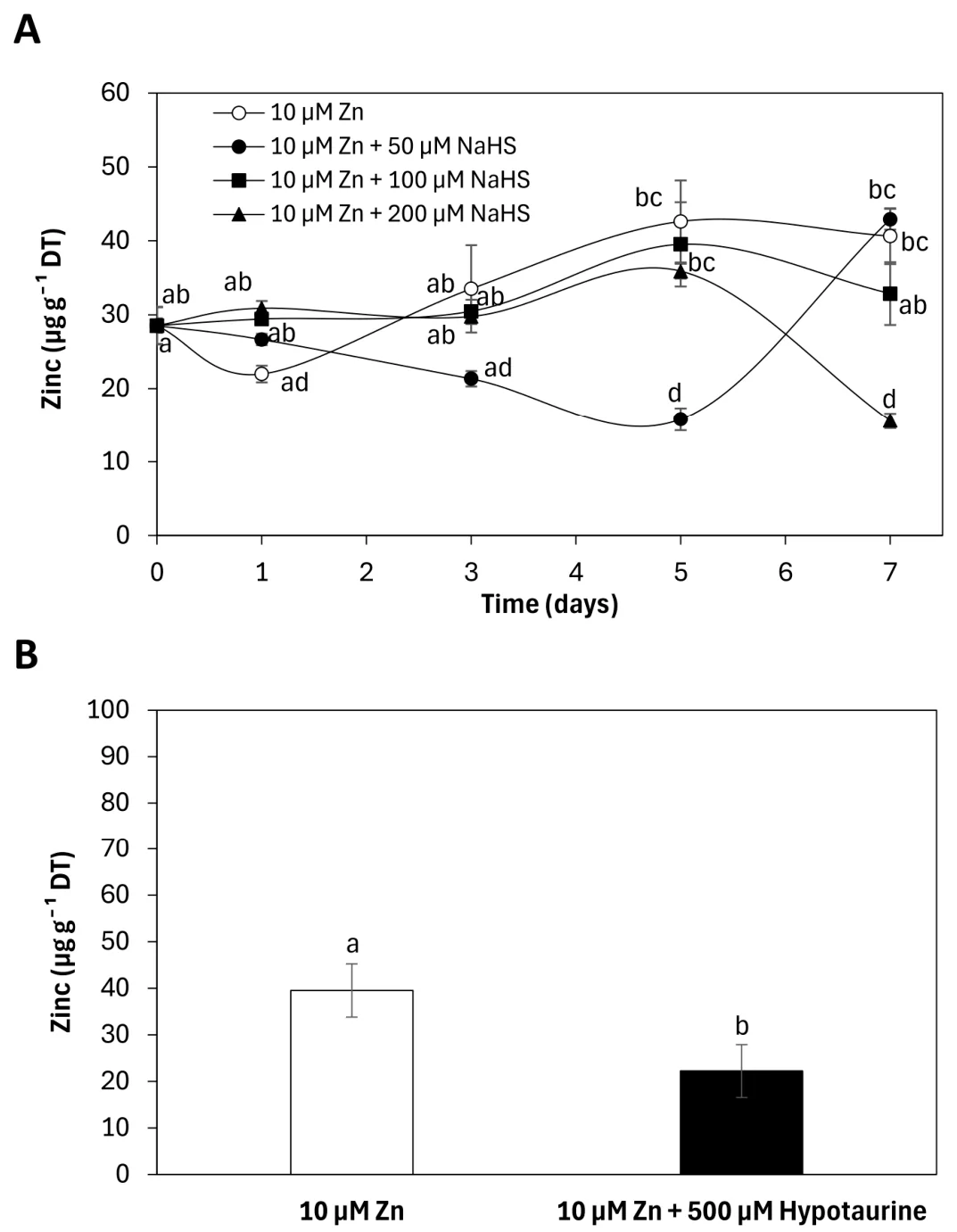
Level of intracellular Zn in the marine alga *U. compressa* cultivated with 10 μM of Zn (open circles), 10 μM of Zn and 50 μM of NaHS (black circles), 10 μM of Zn and 100 μM of NaHS (black squares) or 10 μM of Zn with 200 μM of NaHS (black triangles) for 0 to 7 days (**A**). Level of intracellular Zn in the alga cultivated with 10 μM of Zn (white bar) or with 10 μM of Zn and 500 μM of hypotaurine (black bar) for 5 days. The level of intracellular Zn is expressed as micrograms per gram of dry tissue (DT). Symbols and bars represent mean values ± SD. Significant differences are indicated by letters (*p* < 0.05) (**B**).

The alga treated with 10 μM of Zn for 0 to 7 days showed a basal level of intracellular Zn of 23 μg g^−1^ of dry tissue (DT) that increased to 34 μg g^−1^ of DT at day 3, increased again to 42 μg g^−1^ of DT at day 5 and remained unchanged until day 7 (Figure 1A). The alga treated with 10 μM of Zn and 50 μM of NaHS showed an intracellular level of Zn of 29 μg g^−1^ of DT that decreased to 16 μg g^−1^ of DT at day 5 and increased to the control level at day 7 (Figure 1A). The alga treated with 10 μM of Zn and 100 μM of NaHS showed an intracellular level of Zn of 29 μg g^−1^ of DT at day 1, increased to 40 μg g^−1^ of DT at day 5 and decreased to 33 μg g^−1^ of DT at day 7 (Figure 1A). The alga treated with 10 μM of Zn and 200 μM of NaHS showed that the intracellular level of Zn was similar to the control treated only with Zn until day 5, but it decreased to 16 μg g^−1^ of DT at day 7 (Figure 1A). Thus, the sulfur donor NaHS decreased the level of intracellular Zn, compared to the control treated only with Zn, in *U. compressa*.

The alga cultivated with 10 μM of Zn showed an intracellular level of 39 μg g^−1^ of DT and the alga cultivated with 10 μM of Zn and 500 μM of hypotaurine showed a decrease in Zn level to 22 μg g^−1^ of DT. Thus, this concentration of the sulfur acceptor decreased the intracellular level of Zn in *U. compressa*.

### 2.2. Level of GSH and PCs in Response to Zn and a Sulfur Donor

**Figure 2:**
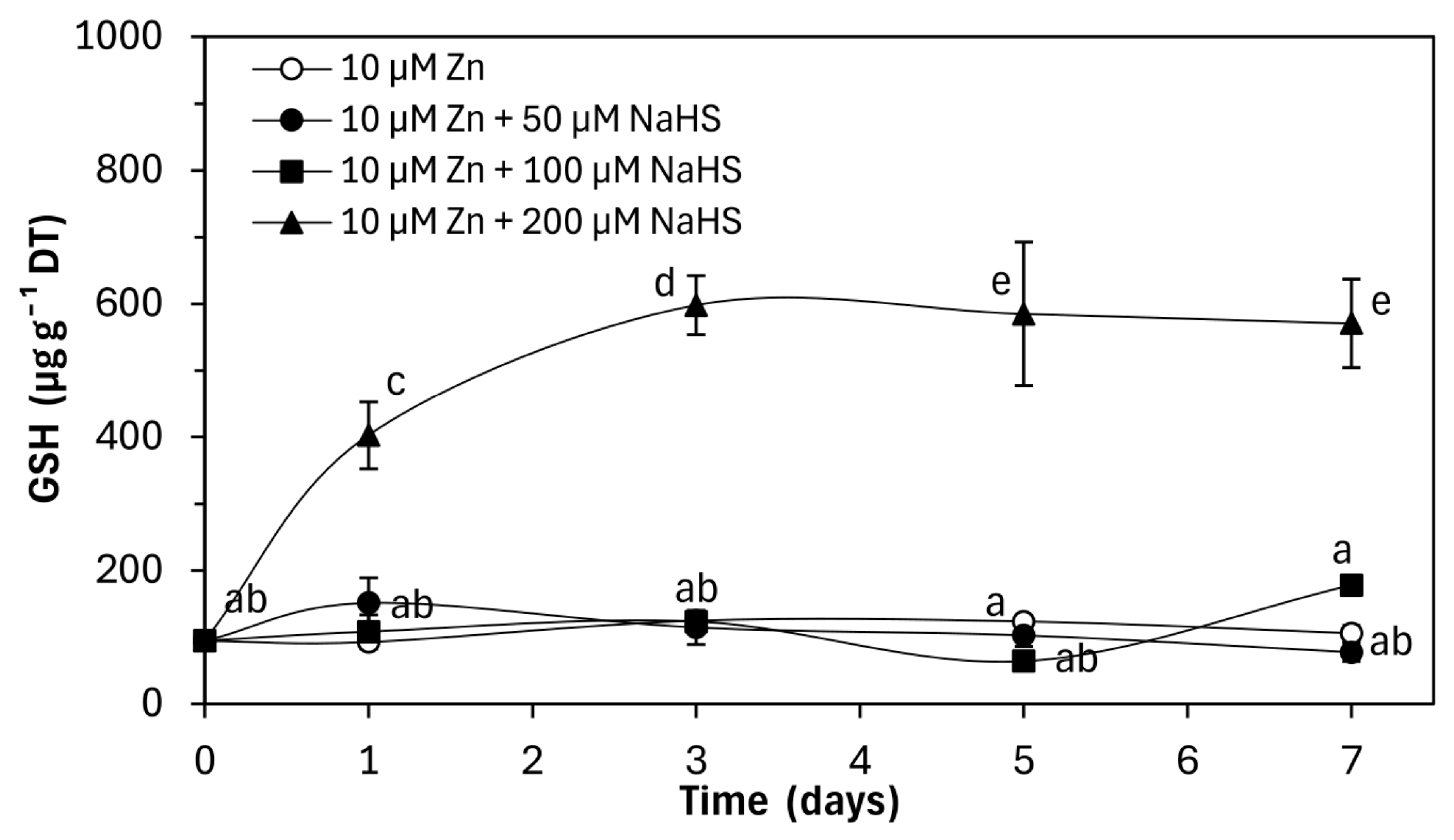
Level of glutathione (GSH) in the marine alga *Ulva compressa* cultivated with 10 μM of Zn (open circles), 10 μM of Zn and 50 μM of NaHS (black circles), 10 μM of Zn and 100 μM of NaHS (black squares), 10 μM of Zn and 200 μM of NaHS (black triangles) for 0 to 7 days. The level of intracellular GSH is expressed as micrograms per gram of dry tissue (DT). Symbols represent mean values ± SD. Significant differences are indicated by letters (*p* < 0.05).

The level of GSH in the alga treated with 10 μM of Zn was 95 μg g^−1^ of DT and this level did not change in the alga treated with 10 μM of Zn and 50 μM of NaHS or 100 μM of NaHS (Figure 2). In contrast, the level of GSH in the alga treated with 10 μM of Zn and 200 μM of NaHS showed an increase in GSH level to 390 μg g^−1^ of DT at day 1 and to 585 μg g^−1^ of DT at day 3 and remained unchanged until day 7 (Figure 2). Thus, only the higher concentration of the sulfur donor NaHS increased the level of GSH, and the GSH level did not parallel the decrease in Zn accumulation in response to a sulfur donor.

**Figure 3:**
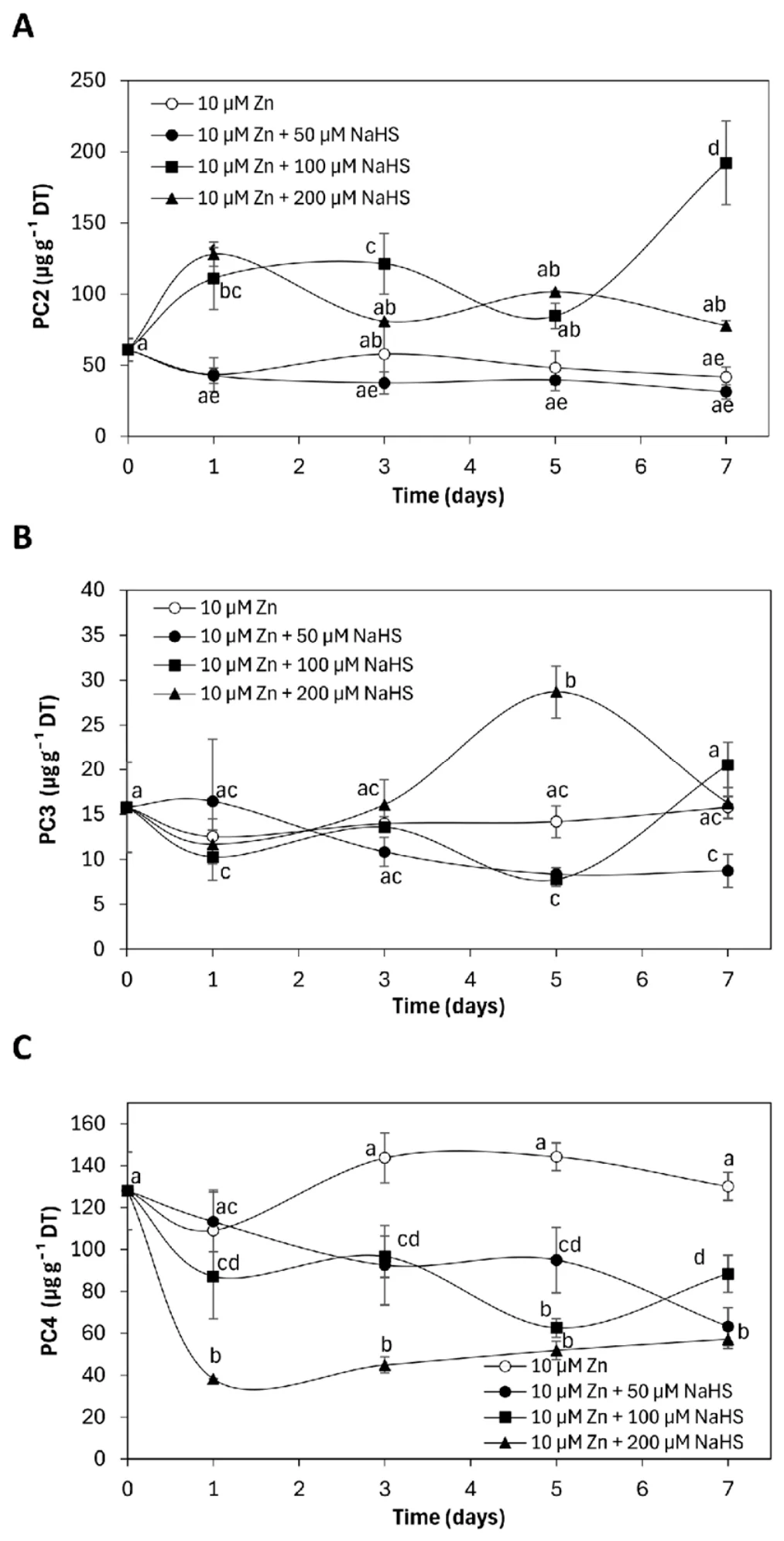
Level of phytochelatins (PCs) corresponding to PC2 (**A**), PC3 (**B**) and PC4 (**C**) in the marine alga *Ulva compressa* cultivated with 10 μM of Zn (open circles), 10 μM of Zn and 50 μM of NaHS (black circles), 10 μM of Zn and 100 μM of NaHS (black squares), 10 μM of Zn and 200 μM of NaHS (black triangles) for 0 to 7 days. The levels of intracellular PCs are expressed as micrograms per gram of dry tissue (DT). Symbols represent mean values ± SD. Significant differences are indicated by letters (*p* < 0.05).

The level of PC2 in the alga treated with 10 μM of Zn was 61 μg g^−1^ of DT at day 0 and remained unchanged until day 7. The level of PC2 in the alga treated with 10 μM of Zn and 50 μM of NaHS decreased to 43 μg g^−1^ of DT at day 1 and remained unchanged until day 7 (Figure 3A). The level of PC2 in the alga treated with 10 μM of Zn and 100 μM of NaHS showed an increase to 111 μg g^−1^ of DT at day 1, decreased to 85 μg g^−1^ of DT at day 5 and increased to 192 μg g^−1^ of DT at day 7 (Figure 3A). The level of PC2 in the alga treated with 10 μM of Zn and 200 μM of NaHS increased to 128 μg g^−1^ of DT at day 1 and it decreased to 81 μg g^−1^ of DT at day 3 and remained unchanged until day 7 (Figure 3A). Thus, the level of PC2 increased in the alga treated with Zn and 100 or 200 μM of NaHS and did not parallel the decrease in Zn accumulation in response to a sulfur donor.

The level of PC3 in the alga treated with 10 μM of Zn was 16 μg g^−1^ of DT at day 0 and it remained unchanged until day 7 (Figure 3B). The level of PC3 in the alga treated with 10 μM of Zn and 50 μM of NaHS remained unchanged until day 3, decreased to 8 μg g^−1^ of DT at day 5 and remained unchanged until day 7 (Figure 3B). The level of PC3 in the alga treated with 10 μM of Zn and 100 μM of NaHS decreased to 8 μg g^−1^ of DT at day 5 and increased to 21 μg g^−1^ of DT at day 7 (Figure 3B). The level of PC3 in the alga treated with 10 μM of Zn and 200 μM of NaHS increased to 25 μg g^−1^ of DT at day 5 and decreased to control level at day 7 (Figure 3B). Thus, the level of PC3 only slightly increased with Zn and 200 μM of NaHS at day 5, and this did not parallel the decrease in Zn accumulation in response to a sulfur donor.

The level of PC4 in the alga treated with 10 μM of Zn was 128 μg g^−1^ of DT at day 0 and it remained unchanged until day 7 (Figure 3C). The level of PC4 in the alga treated with 10 μM of Zn and 50 μM of NaHS decreased to 85 μg g^−1^ of DT at days 3 and 5 and decreased to 63 μg g^−1^ of DT at day 7 (Figure 3C). The level of PC4 in the alga treated with 10 μM of Zn and 100 μM of NaHS decreased to 63 μg g^−1^ of DT at day 5 and increased to 88 μg g^−1^ of DT at day 7 (Figure 3C). The level of PC4 in the alga treated with 10 μM of Zn and 200 μM of NaHS decreased to 38 μg g^−1^ of DT at day 1 and remained unchanged until day 7 (Figure 3C). Thus, the level of PC4 mainly decreased in the alga treated with Zn and 50, 100 or 200 μM of NaHS. It is important to mention that the initial level of PC4 was higher than the level of PC2 and PC3, and the level of PC2 was higher than the level of PC3. In addition, the level of PC2 mainly increased in response to a sulfur donor and the level of PC4 mainly decreased in response to the sulfur donor NaHS, and the level of PC4 did not parallel the decrease in Zn accumulation in response to a sulfur donor.

### 2.3. The Level of MT Transcripts in Response to Zn and a Sulfur Donor

**Figure 4:**
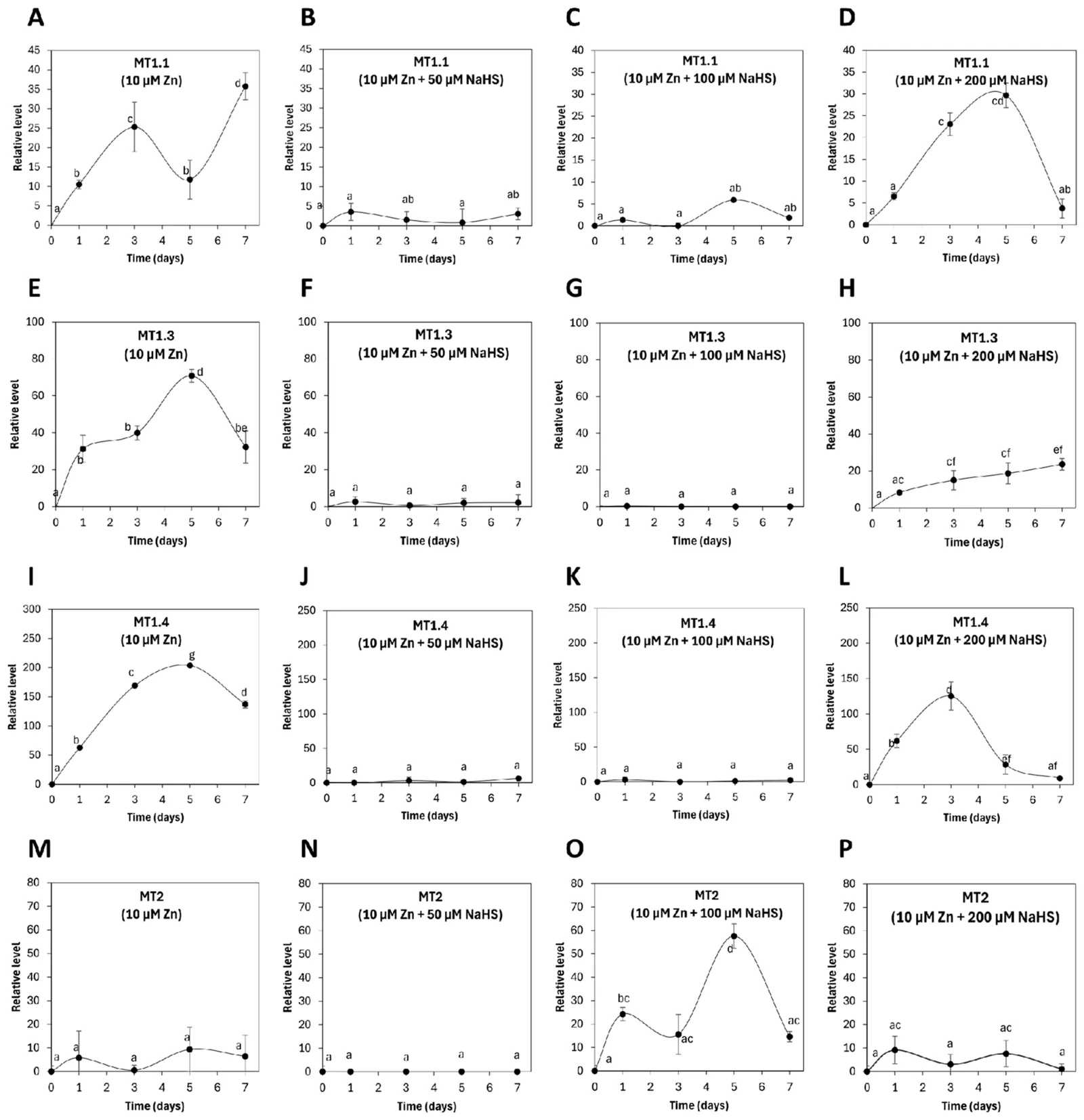
Levels of transcripts encoding metallothioneins (MTs) corresponding to MT1.1 (**A**–**D**), MT1.3 (**E**–**H**), MT1.4 (**I**–**L**) and MT2 (**M**–**P**) in the marine alga *Ulva compressa* cultivated with 10 μM of Zn (**A**,**E**,**I**,**M**), with 10 μM of Zn and 50 μM of NaHS (**B**,**F**,**J**,**N**), with 10 μM of Zn and 100 μM of NaHS (**C**,**G**,**K**,**O**), or with 10 μM of Zn and 200 μM of NaHS (**D**,**H**,**L**,**P**) for 0 to 7 days. The levels of MTs are expressed as 2^−ΔΔCt^. Symbols represent mean values ± SD. Significant differences are indicated by letters (*p* < 0.05).

The relative level of transcripts encoding MT1.1 in the alga treated with 10 μM of Zn increased 11 times at day 1, increased again to 25 times at day 3, decreased to 12 times at day 5, and then increased again to 36 times at day 7 (Figure 4A). The level of transcripts encoding MT1.1 in the alga treated with 10 μM of Zn and 50 and 100 μM of NaHS remained unchanged until day 7 (Figure 4B,C). The level of transcripts encoding MT1.1 in the alga treated with 10 μM of Zn and 200 μM of NaHS increased 23 times at day 3, increased again to 30 times at day 5 and decreased to 4 times at day 7 (Figure 4D). Thus, the level of transcripts encoding MT1.1 mainly increased in response to Zn, and Zn with 200 μM of NaHS, and this transcript level did not parallel the decrease in Zn accumulation in response to a sulfur donor.

The level of transcripts encoding MT1.2 did not increase in response to Zn, or Zn with 50, 100 or 200 μM of NaHS, and this transcript level did not correlate with the decrease in Zn accumulation in response to a sulfur donor.

The level of transcripts encoding MT1.3 in the alga treated with 10 μM of Zn increased 31 times at day 1, increased again to 71 times at day 5 and then decreased to 32 times at day 7 (Figure 4E). The level of transcripts encoding MT1.3 did not increase in the alga treated with 10 μM of Zn and 50 μM or 100 μM of NaHS (Figure 4F,G). The level of transcripts encoding MT1.3 in the algae treated with 10 μM of Zn and 200 μM of NaHS increased 8 times at day 1, 15 times at day 3, 19 times at day 5 and 24 times at day 7 (Figure 4H). Thus, the level of transcripts encoding MT1.3 increased only with Zn, and Zn with 200 μM of NaHS, but mainly with Zn without NaHS. This transcript level did not parallel the intracellular Zn accumulation because the level of Zn decreased in response to Zn and 50, 100 or 200 μM of NaHS.

The level of MT1.4 in the alga treated with 10 μM of Zn increased 63 times at day 1, increased to 170 times at day 3, increased again to 204 times at day 5 and decreased to 137 times at day 7 (Figure 4I). The level of transcripts encoding MT1.4 in the alga treated with 10 μM of Zn with 50 or 100 μM of NaHS did not show an increase until day 7 (Figure 4J,K). The level of transcripts encoding MT1.4 in the alga treated with 10 μM of Zn and 200 μM of NaHS increased 62 times at day 1, 125 times at day 3 and then decreased to 28 times at day 5 and decreased again to 9 times at day 7 (Figure 4L). Thus, the level of transcripts encoding MT1.4 increased only with Zn and Zn with 200 μM of NaHS, and this transcript level did not parallel Zn accumulation because the level of Zn decreased in response to Zn and 50, 100 or 200 μM of NaHS.

The level of transcripts encoding MT2 in the alga treated with 10 μM of Zn did not increase until day 7; similarly, the levels in the alga cultivated with 10 μM of Zn and 50 μM or 200 μM of NaHS did not increase (Figure 4M,N,P). In contrast, the level of transcripts encoding MT2 in the alga treated with 10 μM of Zn and 100 μM of NaHS increased 24 times at day 1, decreased to 16 times at day 3 and increased to 58 times at day 5 and decreased to 15 times at day 7 (Figure 4O). Thus, the level of transcripts encoding MT2 increased only with 10 μM of Zn and 100 μM of NaHS, and not in the other conditions. In contrast, the level of transcripts encoding MT3 did not increase under any of the conditions tested. Thus, the level of transcripts encoding MT2 and MT3 did not parallel the accumulation of intracellular Zn because the level of Zn decreased in response to Zn and 50, 100 or 200 μM of NaHS. Therefore, the expression of MTs in response to 50, 100 or 200 μM of NaHS did not parallel the level of intracellular Zn accumulation in *U. compressa*.

### 2.4. Zn Is Co-Released with GSH to the Culture Medium and This Process Is Not Altered by a Sulfur Donor

**Figure 5:**
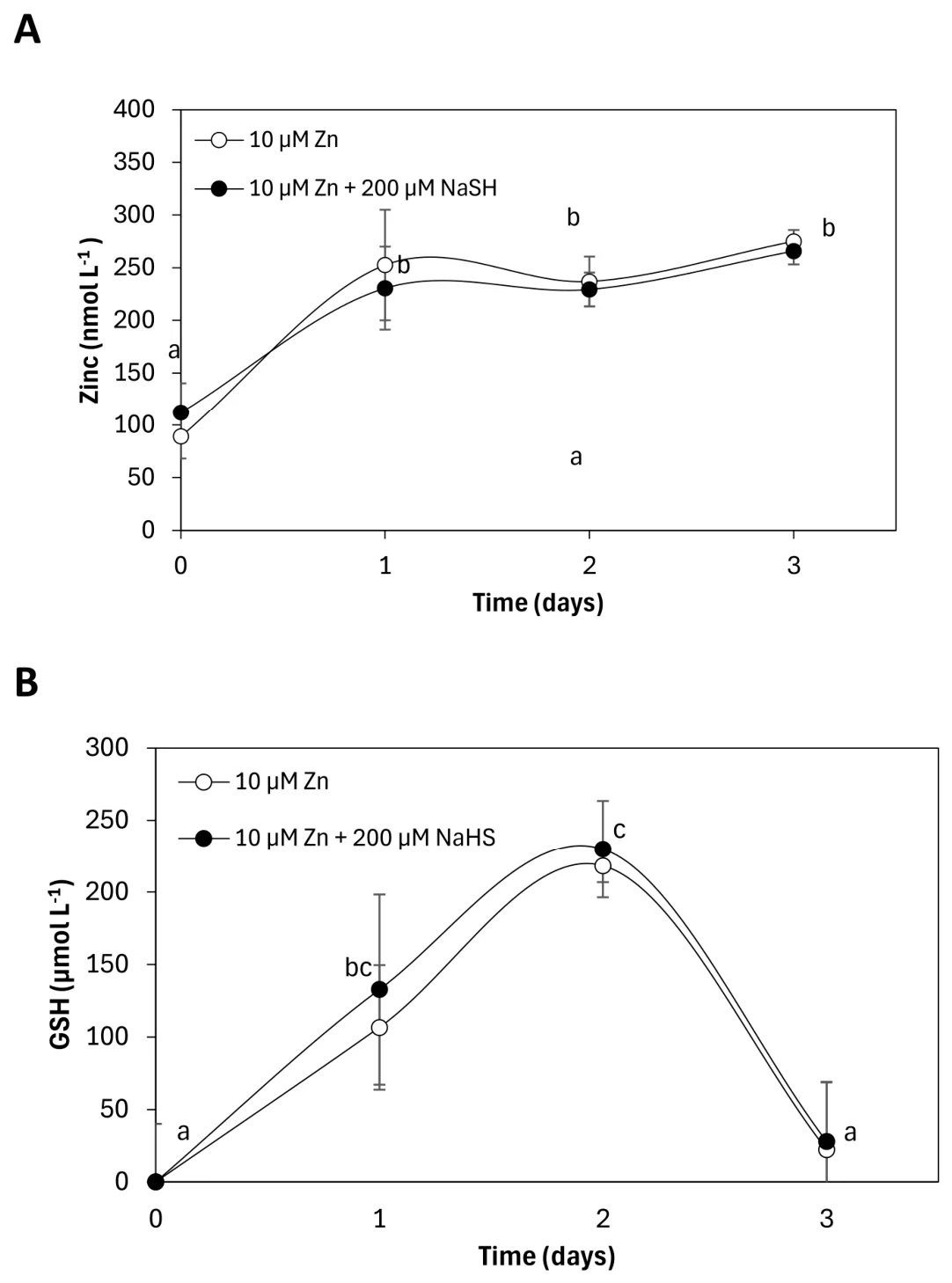
Level of zinc (**A**) and glutathione (**B**) in the culture medium in the alga *Ulva compressa* cultivated with 10 μM of Zn (open circles) or with 10 μM of Zn and 200 μM of NaHS (black circles) for 0 to 3 days. The level of Zn is expressed in nanomoles per liter and the level of GSH is expressed in micromoles per liter. Symbols represent mean values ± SD. Significant differences are indicated by letters (*p* < 0.05).

The alga was cultivated for 5 days and then transferred to a culture medium without Zn, and the level of Zn was analyzed in the culture medium. The level of Zn was 107 nmol L^−1^ at day 0; it increased to 218 nmol L^−1^ at day 1 and remained stable until day 3 (Figure 5A). Thus, Zn is released to the culture medium by the marine alga *U. compressa*. The level of Zn did not increase in response to Zn and 200 μM of NaHS (Figure 5A).

In addition, GSH and PC levels were analyzed in the culture medium. The level of GSH was 0 μmol L^−1^ at day 0; it increased to 107 μmol L^−1^ at day 1, increased again to 218 μmol L^−1^ at day 2, and then decreased to 22 μmol L^−1^ at day 3 (Figure 5B). The level of GSH in the alga cultivated with zinc and 200 μM of NaHS did not change in response to NaHS (Figure 5B). Thus, zinc is co-released with GSH to the culture medium and their release is not increased by a sulfur donor. PCs were not detected in the culture medium. Thus, only Zn and GSH are co-released to the culture medium in *U. compressa*.

## 3. Discussion

The level of intracellular Zn decreased in response to increasing concentrations of a sulfur donor (NaHS) compared to the control treated only with Zn. These results contrast with those previously obtained in this marine alga regarding the accumulation of Cu and Cd since their intracellular accumulation increased in response to NaHS. The level of intracellular Zn in the alga cultivated with 10 μM of Zn was 42 μg g^−1^ of DT at day 5, which was higher than Cd accumulation which was 2.9 μg g^−1^ of DT at day 5 in the alga treated with 10 μM of cadmium for 5 d [19], and this level was lower than that in the alga treated with 10 μM of copper, around 70 μg g^−1^ of DT [18]. These results initially suggest that the alga may release Zn more efficiently to the culture medium than Cu. In fact, *U. compressa* collected in heavy metal-contaminated sites in Northern Chile showed intracellular levels of Cu higher than those of Zn [16]. Interestingly, Cu is accumulated as CuS; Cd is not accumulated as CdS; and Zn is probably not accumulated as ZnS in the alga treated with 10 μM of Cu, Cd or Zn. This phenomenon may be explained by dissociation constants of CuS, CdS and ZnS, since CuS is much more insoluble than CdS, and CdS is much more insoluble than ZnS [20]. Surprisingly, the sulfur acceptor hypotaurine decreased the level of intracellular Zn, but this phenomenon needs to be further studied to be interpreted. Thus, a sulfur donor and a sulfur acceptor decreased the amount of intracellular Zn in *U. compressa*.

It is important to mention that Zn can be accumulated complexed with nicotianamine (NA), a non-proteinogenic metal-chelating amino acid synthesized by nicotianamine synthase (NAS) by condensation of three molecules of S-adenosylmethionine (SAM) [21,22]. In this sense, it has been shown that the overexpression of barley NAS in tobacco and *A. thaliana* increased the amount of Zn and iron (Fe) in seeds of both plants [23]. NAS from *Malus xianojinensis* overexpressed in *A. thaliana* induced an increase in the NA level, as well as in chlorophyll, Fe, Zn and manganese (Mn) levels [24]. In addition, the overexpression of barley NAS in rice increased 1.5- and 2.5-fold, respectively, in the shoots [25]. Moreover, citric acid (CA) can also bind Fe and Zn. It has been shown for citrate synthase (CS) 1 of *M. xiaojinensis* (MxCS1) overexpressed in tobacco that induced the increased CA level and also, Fe, Mn, Cu and Zn [26]. Furthermore, CS of *Malus domestica* (MdCS1) overexpressed in *A. thaliana* induced an increase in CA content, fresh weight, chlorophyll and Fe levels [27]. On the other hand, ferritin is a protein that binds mainly Fe but can also bind Zn, increasing Fe and Zn levels in the leaves [21]. The overexpression of soybean ferritin and rice NAS in rice increased the level of Zn and Fe in the seeds [28]. Thus, there are several mechanisms of Zn binding, other than those using GSH, PCs or MTs, that allow the accumulation of Zn in plants. Similar mechanisms may exist in algae and therefore, it is possible that Zn is accumulated as Zn-NA, Zn-CA or Zn-ferritin in *U. compressa,* but the latter needs to be further explored.

Regarding GSH and Zn accumulation, the level of GSH did not increase in response to 10 μM of Zn or 10 μM Zn with 50 and 100 μM of NaHS, but it increased with Zn and 200 μM of NaHS. This accumulation can be explained by the fact that 200 μM of NaHS increases the activities of GSH-synthesizing enzymes. Regarding PC levels and Zn accumulation, the level of PC2 increased in the alga treated with Zn and 100 or 200 μM of NaHS, the level of PC3 only slightly increased with Zn and 200 μM of NaHS at day 5, and the level of PC4 mainly decreased in the alga treated with Zn and 50, 100 or 200 μM of NaHS. The intracellular PC profiles did not show a pattern corresponding to net intracellular Zn accumulation, suggesting that Zn is probably only released with GSH, and not with PCs, to the culture medium, as in the case of copper [18].

Regarding the level of MTs and Zn accumulation, the level of transcripts encoding MT1.1 mainly increased in response to Zn, and Zn with 200 μM of NaHS. In contrast, the level of transcripts encoding MT1.2 did not increase in response to Zn or Zn with increasing concentrations of NaHS. The level of MT1.3 and the level of MT1.4 increased only with Zn and Zn with 200 μM of NaHS. The level of MT2 only increased with Zn and 100 μM of NaHS whereas the level of MT3 was undetectable in all conditions. The transcript levels of MTs did not parallel the levels of intracellular Zn that decreased in response to Zn and increasing concentrations of NaHS and, thus, MTs may not be implicated in Zn accumulation. However, the levels of MTs proteins were not detected in this work, and they could be analyzed in the future. It is interesting to mention that the expression of MTs in the alga cultivated with 10 μM Zn, 10 μM of Cd and 10 μM of Cu with increasing concentrations of NaHS differentially responds to these heavy metals and a sulfur donor. For example, the most highly expressed MT in response to Cu and 100 μM of NaHS was MT1.2 that increased 80 times [18]; the most highly expressed MT in response to Cd and 50 μM of NaHS was also MT1.2 that increased 600 times [19] and the most highly expressed MT in response to Zn and 200 μM of NaHS was MT1.4 that increased 125 times. Thus, the expression of MTs in the marine alga *U. compressa* differentially responds to the same micromolar concentrations of Cu, Cd and Zn.

Regarding Zn, GSH and PCs released to the culture medium, it was shown that Zn was co-released only with GSH, and not with PCs, and that NaHS did not increase the levels of Zn or GSH in the culture medium. The results are in accord with those obtained for Cu and Cd that are released mainly with GSH [18,19], but Cd is released also with PCs [19]. In contrast, the level of Zn and GSH did not show a higher increase in response to 200 μM of NaHS, but the level of Cd did increase in the culture medium in response to NaHS [19]. The release of Cu and GSH to the culture medium in response to NaHS has not been analyzed until now. Thus, Zn, Cu and Cd are co-released to the culture medium with GSH, but Zn release is unchanged in response to a sulfur donor, as in the case of Cd [19]. This metal release suggests that Zn transporters are not susceptible to persulfidation induced by a sulfur donor, as was proposed for Cd release transporters [19].

## 4. Materials and Methods

### 4.1. Sampling of Ulva compressa

*U. compressa* was collected in Cachagua (32°34′ S) located in central Chile, from April to November 2025. Algae were placed in a cooler with ice, transported to the laboratory and cleaned manually. *U. compressa* collected in Cachagua and cultivated with 10 µM of copper was subjected to a transcriptomic analysis and the bioinformatic analysis of chloroplastic TuFA, PsbA and PsaA, corresponding to sequences PZ492934, PZ492935 and PZ492936, respectively, showed a 98–100% nucleotide similarity to sequences present in the NCBI database under Gene IDs 59422245, 59422242 and 59422235, respectively, indicating that the Chilean alga is indeed *U. compressa*. Algae were sonicated twice for 2 min using an ultrasound bath (Hilab Innovation Systems, model SK221, Jilin, China) to remove epiphytic bacteria. Algae were maintained in aerated synthetic seawater prepared with 35 g of sea salts in 1 L of distilled water in a culture chamber at 14 °C and with a light period of 12 h light/12 h darkness.

### 4.2. Culture of U. compressa with Zn, NaHS or Hypotaurine

Algae (10 g of fresh tissue (FT)) were cultivated in 300 mL of synthetic seawater with 10 µM of Zn (control), and 10 µM of Zn supplemented with 50, 100 or 200 µM of NaHS for 1, 3, 5 and 7 days or with 500 µM of hypotaurine for 5 days. Algae were cultivated in aerated seawater, in triplicate, in a culture chamber at 14 °C, and with a light period of 12 h of light/12 h of darkness and pH 8. After culture, algae were washed twice with 60 mL of 50 mM of Tris-HCl-10 mM EDTA to remove Zn from cell walls of the alga, to detect only intracellular Zn. Algae (9 g of FT) were dried in an oven at 60 °C until they reached a constant weight (700–800 mg of dry tissue (DT)) and the levels of intracellular Zn, GSH and PCs were quantified. Algae (1 g of FT) were used to quantify transcripts encoding MTs.

### 4.3. Quantification of Intracellular Zn in U. compressa

Quantification of intracellular Zn was performed as described in [18]. Algae (500 mg of DT) were incubated in 9 mL of 65% nitric acid and 1 mL of 30% hydrogen peroxide in Teflon vials at 1800 W for 20 min to reach 210 °C and for 15 min at 210 °C. When the temperature reached 30 °C, the vials were placed at 4 °C for 20 min. The vials were then opened and the final volume was adjusted to 10 mL with distilled water. The level of intracellular Zn was determined using atomic absorption spectroscopy (Analytik Jena, model Nova350, Jena, Germany) and the calibration curve was prepared with aqueous solutions containing 0.5, 1, 2, 3, 5 and 7 µg mL^−1^ of Zn.

### 4.4. Extraction and Quantification of GSH and PCs

Extraction and quantification of GSH and PCs were performed as described in [18]. Algae (200 mg of DT) were frozen in liquid nitrogen and homogenized in a mortar with a pestle. Then, 1.2 mL of 0.1% of trifluoroacetic acid (TFA)-6.3 mM diethylenetriamine penta-acetic acid (DTPA) was added, and homogenization was pursued until thawing. The mixture was centrifuged at 14,000 rpm for 20 min and the supernatant was recovered. The supernatant was filtered through 0.22 µm PVDF filters and an aliquot of 25 µL was mixed with 45 µL of 200 mM HEPES (pH = 8)-6.3 mM DTPA and 1 µL of 25 mM of monobromobimane and incubated at room temperature, in darkness, for 30 min. The reaction was stopped by addition of 30 µL of 1 mM methanesulfonic acid.

The levels of GSH and PCs was determined using High-Performance Liquid Chromatography (HPLC) equipment (Agilent, model 1260, Infinity, Santa Clara, CA, USA) and data were obtained using Open-Lab software, version A.01.04.218. An aliquot of 20 µL was analyzed using a reverse-phase C-18 column having a 5 µm particle size, 4.6 mm internal diameter and 15 cm length (Agilent, Santa Clara, CA, USA) at 24 °C. GSH and PCs were eluted with solvent A (0.1% TFA as aqueous solution) and solvent B (100% acetonitrile) and linear gradient of 10 min from 0 to 20% of solvent B, 10 min from 35 to 100% of solvent B, at a flow rate of 1 mL min^−1^. Pure GSH, PC2, PC3 and PC4 were dissolved in distilled water and used as standards and they showed retention times of 9, 10, 13 and 16 min. GSH and PCs were detected using a fluorescence detector with an excitation wavelength of 380 nm and an emission wavelength of 470 nm. GSH and PCs were quantified using a calibration curve for each compound of 1 to 50 µM.

### 4.5. Extraction of Total RNA and Quantification of Transcripts Encoding MTs

Extraction of total RNA and quantification of transcripts were performed as described in [18]. Algae (100 mg of FT) were frozen in liquid nitrogen and pulverized in a mortar with a pestle and 700 µL of TRK buffer was added. Total RNA was extracted using an E.Z.N.A. total RNA kit (Omega Biotek, Norcross, GA, USA) and quantified by absorbance at 260 nm using a Tecan microplate reader (Zurich, Switzerland). Transcripts encoding MTs and the housekeeping β-tubulin were amplified using PCR primers described in Table 1.

Synthesis of cDNA was performed using 1 µg of total RNA and iScript reverse transcription mix (BioRad, Hercules, CA, USA); the reaction mixture was brought to a final volume of 20 µL with DEPC-treated water (water-DEPC) and the solution was incubated for 5 min at 25 °C, for 20 min at 46 °C and for 1 min at 95 °C. The amplification of cDNAs encoding MTs and tubulin was performed using 2 µL of cDNAs, 5 µL of SSO advance SYBR green supermix (BioRad, Hercules, CA, USA) and 1 µL of each PCR primer and brought to a final volume of 10 µL with water-DEPC. The amplification cycles were 40 s at 95 °C, 10 s at 55 °C and 30 s at 65 °C using a thermocycler Aria MX (Agilent, Santa Clara, CA, USA).

### 4.6. Analysis of Zn, GSH and PCs Extrusion to the Culture Medium in U. compressa

The amount of Zn in the culture medium was analyzed by electrothermal atomic absorption spectroscopy (ET-AAS) [29]. An aliquot of 0.5 mL was filtered through 0.22 μm PVDF membranes, diluted 50 times with distilled and filtered water and analyzed using ET-AAS (Thermo Fisher Scientific, model iCE300, Waltham, MA, USA, detection limit of 1 ng L^−1^) equipped with a Zn hollow-cathode lamp (Heraeus, Hanau, Germany), with argon as a purge gas at a flow rate of 0.2 mL min^−1^. A 1 mg mL^−1^ standard solution (Merck, Darmstadt, Germany) was used for calibration; the equipment diluted 1 μL of the standard with 50 mL of Milli-Q water, and 10 mg mL^−1^ of magnesium nitrate was added as a matrix modifier. The calibration curve was performed using solutions containing 0, 3, 6, 13 and 20 μg L^−1^ with an R^2^ of 0.98.

Algae (10 g) were cultivated in 300 mL of artificial seawater with 10 µM of Zn for 5 days in triplicate; they were then transferred to seawater without Zn and cultivated for 1, 2 and 3 days. Algae were dried in an oven at 60 °C until they reached a constant weight, and the amounts of GSH and PCs were analyzed as described in Section 4.4.

### 4.7. Statistical Analyses

Significant differences were determined by two-way ANOVA followed by using Tukey’s multiple-comparison test, Shapiro–Wilk normality test and software GraphPad Prism 8. Differences among mean values were considered significant at a probability of 5% (*p* < 0.05).

## 5. Conclusions

In this work, we showed that a sulfur donor (NaHS) decreased the intracellular accumulation of Zn and a sulfur acceptor (hypotaurine) also decreased the intracellular level of Zn. The levels of GSH and PCs, as well as the expression of MTs did not correlate with intracellular Zn accumulation in response to a sulfur donor. In addition, Zn was co-released to the culture medium with GSH, and not with PCs, suggesting that the excess of Zn is mainly extruded from the alga with GSH, and not accumulated as ZnS. Zn and GSH were co-released to the culture medium, and their levels did not change in response to NaHS, which contrasts with the co-release of Cd with GSH that increased in response to NaHS.

## Figures and Tables

**Table 1:** PCR primers for amplification of tubulin and MTs.

Tubulin	5′-TGCAACTTTTGTAGGCAACTC-3′	5′-CAGTGAACTCCATCTCGTCC-3′
MT1.1	5′-CATGGACTGCCGTTGCG-3′	5′-AGCTAGCACTTGCAACCG C-3′
MT1.2	5′-CATCATGGATTGCCGCTG-3′	5′-ATCAGCAGCAGCAGCAGC-3′
MT1.3	5′-CATGGACTGCCGTTGCG-3′	5′-AGCTAGTGCGAGTGAGCGC-3′
MT1.4	5′-CATGGACTGCCGTTGCG-3′	5′-CATCTAGCGCGAGCGAGC-3′
MT2	5′-ATGGACTGCCGTTGCGAC-3′	5′-TCTTGCAGGCGCAGGTG-3′
MT3	5′-TCTTGTTGTGAAGCCAGTGA-3′	5′-CACAGTTGCATTCTGCGGTT-3

## Data Availability

https://doi.org/10.6084/m9.figshare.33493714.
